# The Toll Signaling Pathway in the Chinese Oak Silkworm, *Antheraea pernyi*: Innate Immune Responses to Different Microorganisms

**DOI:** 10.1371/journal.pone.0160200

**Published:** 2016-08-02

**Authors:** Ying Sun, Yiren Jiang, Yong Wang, Xisheng Li, Ruisheng Yang, Zhiguo Yu, Li Qin

**Affiliations:** 1 College of Plant Protection, Shenyang Agricultural University, Shenyang, 110866, China; 2 College of Bioscience and Biotechnology, Liaoning Engineering & Technology Research Center for Insect Resources, Shenyang Agricultural University, Shenyang, 110866, China; 3 Sericultural Research Institute of Liaoning Province, Fengcheng, 118100, China; Institute of Plant Physiology and Ecology, CHINA

## Abstract

The Toll pathway is one of the most important signaling pathways regulating insect innate immunity. Spatzle is a key protein that functions as a Toll receptor ligand to trigger Toll-dependent expression of immunity-related genes. In this study, a novel *spatzle* gene (*ApSPZ*) from the Chinese oak silkworm *Antheraea pernyi* was identified. The *ApSPZ* cDNA is 1065 nucleotides with an open reading frame (ORF) of 777 bp encoding a protein of 258 amino acids. The protein has an estimated molecular weight of 29.71 kDa and an isoelectric point (PI) of 8.53. ApSPZ is a nuclear and secretory protein with no conserved domains or membrane helices and shares 40% amino acid identity with SPZ from *Manduca sexta*. Phylogenetic analysis indicated that ApSPZ might be a new member of the Spatzle type 1 family, which belongs to the Spatzle superfamily. The expression patterns of several genes involved in the Toll pathway were examined at different developmental stages and various tissues in 5th instar larvae. The examined targets included *A*. *pernyi spatzle*, *GNBP*, *MyD88*, *Tolloid*, *cactus* and *dorsalA*. The RT-PCR results showed that these genes were predominantly expressed in immune-responsive fat body tissue, indicating that the genes play a crucial role in *A*. *pernyi* innate immunity. Moreover, *A*. *pernyi* infection with the fungus *Nosema pernyi* and the gram-positive bacterium *Enterococcus pernyi*, but not the gram-negative bacterium *Escherichia coli*, activated the Toll signaling pathway. These results represent the first study of the Toll pathway in *A*. *pernyi*, which provides insight into the *A*. *pernyi* innate immune system.

## Introduction

The Toll pathway is one of the most important signaling pathways regulating insect innate immunity. Various studies have shown that Toll signaling plays a crucial role in insect innate immunity to microbial infections in flies [1], silkworm [2], and tobacco hornworm [3]. It has been shown that the Toll pathway mediates the production of antimicrobial peptides in response to infection with gram-positive bacteria or fungi. Moreover, Toll signaling is important to the antiviral response and is required for efficient inhibition of *Drosophila* X virus replication and for resistance to oral infection with the *Drosophila* C virus in *Drosophila* [4,5].

However, there is limited information on the Toll signaling pathway in *Antheraea pernyi*. The Chinese oak silkworm (*Antheraea pernyi* Guérin-Méneville, 1855; Lepidoptera: Saturniidae) is a well-known wild silkworm used for insect food and silk production. Chinese farmers developed rearing methods for the Chinese oak silkworm approximately 400 years ago [6]. Currently, the Chinese annual output of tussah cocoons is approximately 8×10^4^ t, which is nearly 90% of the total output of wild silk worldwide, and the income from tussah rearing has become the main economic source in many sericultural areas. There are approximately one hundred twenty tussah varieties in China, and they can be divided into four races based on larval skin color: yellow, yellow-cyan, white, and blue [7]. Currently, the products from *A*. *pernyi*, such as silk, pupae and moths, are used in many fields. For example, tussah silk fibroin nanoparticles have been used as a sustained drug delivery vehicle [8], and tussah pupae homogenates were used to enhance the gelation properties of surimi from yellowtail seabream [9]. Therefore, the use of tussah products is common and wide-ranging. With new developments in biotechnology, more attention has been paid to the functional genes of *A*. *pernyi*, and several genes from *A*. *pernyi* have been isolated and characterized [10,11].

There are significant differences between the domestic silkworm (*Bombyx mori*) and the Chinese oak silkworm (*A*. *pernyi*). Unlike the domestic silkworm, *A*. *pernyi* larvae are fed on the leaves of oak trees in tussah-feeding oak forests until cocooning during the larval stage. Therefore, there is a high risk of *A*. *pernyi* larvae infection by different microorganisms in the wild. Moreover, substantial economic losses in tussah production are associated with different diseases every year. However, it is evident that *A*. *pernyi* must have immune responses to defend against different microorganisms, as tussah production has lasted for hundreds of years. Different developmental stages of *A*. *pernyi* and survival conditions of *A*. *pernyi* larvae are shown in S1 and S2 Figs.

Insects possess an innate immune system that responds to invading microorganisms. In recent years, immune response-related genes have become an important focus of *A*. *pernyi* research. Fifty immune response-related genes and ten stress response genes were identified from a subtractive cDNA library in *A*. *pernyi* challenged with *Escherichia coli* [12]. Three small heat shock proteins (sHSPs) encoding HSP21, HSP21.4 and HSP20.8 (named as *Ap-sHSP*21, *Ap-sHSP*21.4 and *Ap-sHSP*20.8, respectively) were isolated from *A*. *pernyi*. Further studies have shown that these sHSPs might play important roles in *A*. *pernyi* upon challenge with different microorganisms or under stress conditions [13–15]. Expression of an apolipophorin-III (*ApoLp-III*) gene from *A*. *pernyi* pupae (*Ap-ApoLp-III*) was significantly up-regulated in response to different microorganisms, and RNA interference showed that *Ap-ApoLp-III* might function in the *A*. *pernyi* innate immune system [16].

Previous studies of *A*. *pernyi* innate immunity have mainly focused on the prophenoloxidase (pro-PO) system. It has been reported that lectin increases in response to the intrusion of foreign substances in *A*. *pernyi* [17]. In *A*. *pernyi*, The β-1,3-glucan recognition protein (*Ap-βGRP*) and lectin-5 (*Aplectin*-5) were induced by all microorganisms, including *Bacillus subtilis*, *E*. *coli*, *Antheraea pernyi* nuclear polyhedrosis virus (ApNPV) and *Saccharomyces cerevisiae*, whereas *A*. *pernyi C-type lectin* 1 was not induced by gram-positive bacteria, and the genes exhibited significantly different expression levels in different tissues. The results suggest that lectins might have various functions in different *A*. *pernyi* tissues [18]. A 1,3-β-D-glucan recognition protein from *A*. *pernyi* (*Ap-βGRP*) that specifically binds 1,3-β-D-glucan from yeast but not *E*. *coli* or *Micrococcus luteus* has been identified, and the presence of both 1,3-β-D-glucan and Ap-βGRP triggered the pro-PO system together but not separately [19]. An *A*. *pernyi* C-type lectin (*Ap*-rCTL) involved in the pro-PO activating system plays an important role in *A*. *pernyi* innate immunity as a pattern recognition protein that can recognize and trigger the agglutination of bacteria and fungi [20]. *A*. *pernyi* prophenoloxidase (*ApPPO*) was also cloned, and *ApPPO* expression was significantly up-regulated in *A*. *pernyi* tissues following microbial infection. Recombinant ApPPO is able to kill bacteria and induce the cecropin transcription in larvae [21]. Additionally, many genes coding for immune proteins from *A*. *pernyi* have been cloned, such as hemolin [22], which might affect the progress of viral infection in *A*. *pernyi* [23].

In *A*. *pernyi*, many immune genes involved in the Toll signaling pathway have been isolated, although there is limited information about Toll signaling in this organism. Two Rel/NF-kB-related genes, *Apdorasl*A and *Apdorsal*B, were cloned from *A*. *pernyi*. The cloned genes were differentially expressed in response to different microorganisms, indicating that Apdorsal might be involved in the immune response to viruses, fungi and gram-positive bacteria in *A*. *pernyi* [24]. Although the sequences of many genes involved in the *A*. *pernyi* Toll pathway have been submitted to GenBank, including GNBP (accession number: KF725771), MyD88 (accession number: KF670143), Tolloid (accession number: KF670144), and cactus (accession number: KF670142), there has been no report or record of the *spatzle* gene in *A*. *pernyi* to our knowledge. It is well known that spatzle is a key signal transducer for immune responses, a ligand for Toll receptors and a very important functional protein for activating the Toll pathway in response to different microorganisms.

In this study, a novel *spatzle* gene (*ApSPZ*) from the Chinese oak silkworm, *A*. *pernyi*, was identified while investigating the Toll signaling pathway in response to different microorganisms. Furthermore, the expression patterns of genes involved in the Toll pathway were examined in *A*. *pernyi* infected with different microorganisms. The results of this analysis provide a foundation for further investigation of the Toll signaling pathway in *A*. *pernyi*.

## Materials and Methods

### Sample collection and preparation

*Antheraea pernyi* variety *Shenhuang No*. *2* was used in this study. The eggs (on the fifth day), fifth instar larvae (on the third day), pupae and moths were frozen in liquid nitrogen and stored at –80°C until use. The epidermis, silk glands, blood, gonads, Malpighian tubules, fat body, midgut, and muscle were dissected from fifth instar *A*. *pernyi* larvae, immediately frozen in liquid nitrogen and stored at –80°C until use. All of the samples were used for RT-PCR.

On the first day, fifth instar larvae were orally administered 20 μL of different microorganisms separately suspended in sterile water, including *E*. *coli* (Ec, 1.2×10^7^ bacterial cells/mL), *Enterococcus pernyi* (Ep, 2.0×10^7^ cells/mL), and *Nosema pernyi* (Np, 5.0×10^7^ spores/mL), and larvae fed sterile water were used as controls (CK). Fat bodies dissected from different groups were used for RNA extraction 24 h and 48 h after inoculation and were stored at -80°C for qRT-PCR testing. *A*. *pernyi* larvae were kept in a rearing chamber at 23±2°C with 70±5% relative humidity and were fed fresh *Quercus mongolica* leaves.

### Total RNA extraction and cDNA synthesis

Total RNA was extracted using TRIzol^®^ Reagent (Invitrogen) according to the manufacturer’s protocol. RNA degradation and contamination were monitored on 1% agarose gels. The extracted total RNA was quantified using a NanoDrop 2000 UV-Vis Spectrophotometer (Thermo Scientific, USA). First-strand cDNA synthesis was performed using an M-MuLV First Strand cDNA Synthesis Kit (Sangon Biotech, China). The full-length *A*. *pernyi spatzle* cDNA was cloned using reverse transcription PCR, 5' RACE and 3' RACE. RACE was performed using a 5' RACE system (version 2.0, Invitrogen) and a SMARTer^™^ RACE cDNA Amplification Kit (Clontech) according to the user manual. The cDNAs derived from all of the samples were used for gene expression analysis.

### RT-PCR analysis

Reverse transcription-polymerase chain reaction (RT-PCR) was used to analyze gene expression patterns. The cDNA samples were used as templates for RT-PCR amplification. RT-PCR was performed with gene-specific primer pairs (shown in S1 Table) for each gene. The *A*. *pernyi actin* gene (GU073316) was used as an internal control to normalize the levels of genes in the Toll pathway using the primers *Apactin*-F (5'-CCAAA GGCCA ACAGA GAGAA GA-3') and *Apactin*-R (5'-CAAGA ATGAG GGCTG GAAGA GA-3') [25]. The total reaction volume was 25 μL, and each reaction contained 10 pmol each primer, 0.25 mM each dNTP, 1× buffer, 2 mM MgCl_2_, 2.5 units *Taq* DNA polymerase (TaKaRa), and normalized amounts of the cDNA template. PCR was performed as follows: an initial 3 min step at 95°C; 28 cycles of 30 sec at 95°C, 30 sec at 55°C, and 30 sec at 72°C; and a final extension period of 10 min at 72°C. The amplified products were detected on a 1.5% agarose gel with ethidium bromide staining, and Quantity One Version 4.6.2 (Bio-Rad, USA) was used to estimate the intensities of the visualized target band for each target gene compared to the *A*. *pernyi actin* gene. RT-PCR experiments were performed three times. The RT-PCR products were purified from the gel and sequenced to confirm the specificity of the RT-PCR amplification.

### Quantitative real-time PCR analysis

To examine the *A*. *pernyi* immune response against different microorganisms, the relative mRNA levels of genes involved in *A*. *pernyi* immunity were evaluated using quantitative real-time PCR (qRT-PCR). The qRT-PCR amplification was performed using SYBR Premix Ex Taq^™^ (TaKaRa, Japan) and a Roche Light Cycler 480 (Hoffmann-La Roche Ltd.). qRT-PCR was performed with the following protocol: initial denaturation at 95°C for 2 min; 40 cycles of 15 s at 95°C, 30 s of annealing at 60°C, and 30 s of extension at 68°C; and a 60–95°C melting curve to analyze the amplified products. The gene-specific primer pairs for qRT-PCR are shown in S1 Table. The *A*. *pernyi* actin gene (GU073316) was amplified as an internal control to normalize the transcript levels of the genes using *Apactin*-qRT-F (5'-ATGTG CAAGG CCGGT TTC-3') and *Apactin*-qRT-R (5'-TTGCT CTGTG CCTCA TCACC-3'). The relative gene expression levels were calculated using the 2^-ΔΔCt^ method [26]. All of the samples were analyzed in triplicate. Experimental data were analyzed using a two-tailed Student’s t test (*P<0.05; **P<0.01).

### Bioinformatics analysis

The cDNA and deduced amino acid sequence analyses were performed using DNASTAR software (DNASTAR Inc., www.dnastar.com). The isoelectric point and molecular weight of the deduced amino acid sequence were predicted using ExPASy (http://www.expasy.org/tools/pi_tool.html). Conserved domains were predicted using NCBI website tools (http://www.ncbi.nlm.nih.gov/Structure/cdd/wrpsb.cgi/) [27]. SignalP tools (http://www.cbs.dtu.dk/services/SignalP/) were used to predict signal peptides [28], and the subcellular localization of the protein was also predicted (http://www.bioinfo.tsinghua.edu.cn/SubLoc/) [29]. Transmembrane protein topological structure was analyzed with TMHMM tools (http://www.cbs.dtu.dk/services/TMHMM/) [30], and a motif scan was performed (http://hits.isb-sib.ch/cgi-bin/motif_scan) [31]. Predictions of N- and O-glycosylated sites were also performed (http://www.cbs.dtu.dk/services/NetNGlyc/ and http://www.cbs.dtu.dk/services/NetOGlyc/) [32]. Homology analysis of the deduced amino acid sequence was performed using the BLAST tool in GenBank (Blastp). Multiple sequence alignments were performed using ClustalX software [33], and the protein structure prediction was performed using http://swissmodel.expasy.org/interactive. The unrooted tree was generated with TreeView Version 1.6.6 [34].

## Results

### Sequence analysis of the *ApSPZ* gene

**Fig 1:**
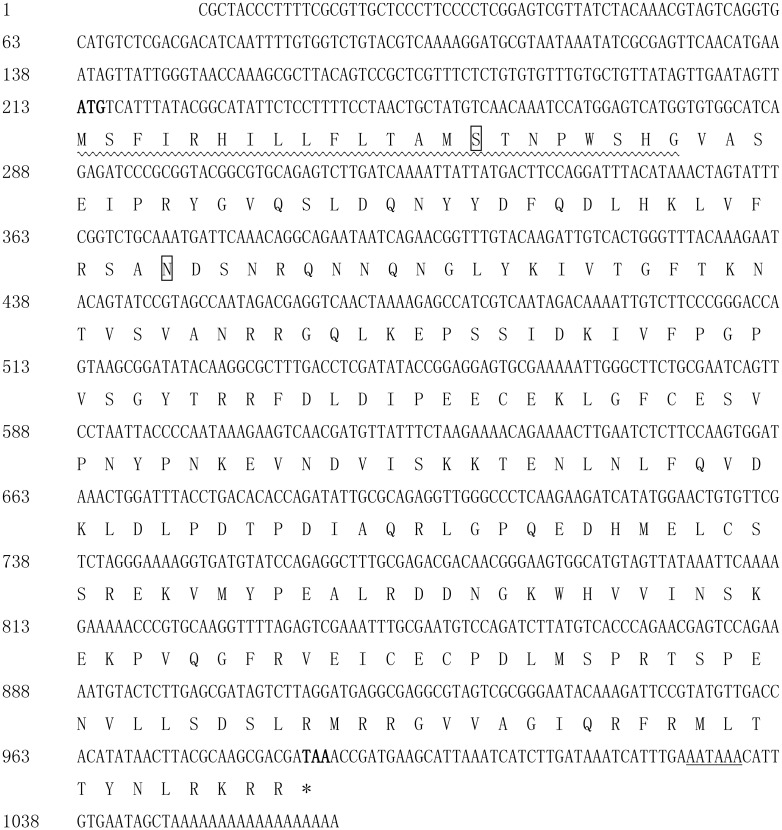
cDNA sequence and deduced amino acid sequence of the *ApSPZ* gene. Nucleotides are numbered on the left of each line. The deduced amino acid sequence is shown below the nucleotide sequence. The ATG initiation codon is in bold, and the TAA termination codon is in bold and marked with an asterisk. The potential polyadenylation AATAAA sequence near the end of the 3′ untranslated region is underlined. The predicted signal peptide is wave underlined. This cDNA sequence has been deposited in GenBank under accession number KU323402.

We performed transcriptome sequencing on the Chinese oak silkworm *A*. *pernyi*, and high-quality reads were deposited in the NCBI SRA database (Accession numbers: SRR2919240, SRR2919241, SRR2919242 and SRR2919243). Assembly of the high quality reads was performed using the Trinity de novo assembly program. A unigene (comp748335_c0) annotated *Manduca sexta* Spz1A (GenBank accession No.GQ249944.1) encoding 256 nucleotides was selected. Based the unigene sequence, a novel *spatzle* gene (*ApSPZ*) from *A*. *pernyi* was first identified using RT-PCR, 5' RACE and 3' RACE. The isolated *ApSPZ* cDNA was 1065 nucleotides with an open reading frame (ORF) of 777 bp that encodes a 258 amino acids protein. The cDNA sequence contains a 212 bp 5' -untranslated region (UTR) and a 76 bp 3'-UTR with a polyadenylation signal sequence (AATAAA) at position 1027 and a poly(A) tail. The initiation codon ATG and the termination codon TAA are at positions 213 and 987, respectively (Fig 1). The predicted molecular weight and isoelectric point (pI) of *Ap*SPZ were 29.71 kDa and 8.53, respectively. ApSPZ was assigned its name because of its similarity to known spatzle proteins. This cDNA sequence has been deposited in GenBank under accession no. KU323402.

No conserved domains were identified for *Ap*SPZ. The topological structural analysis of the trans-membrane protein showed that it contained no membrane helices. Prediction of subcellular localization by SubLoc indicated that the protein is nuclear (reliability index: RI = 5; expected accuracy = 94%). Protein signal peptide analysis showed that the deduced signal peptide cleavage site is between amino acids 22 and 23 (signal peptide probability: 0.601), indicating that *Ap*SPZ is a secretory protein. The predictions of N- and O-glycosylated sites showed that the protein contains an N-glycosylated site at amino acid 54 and an O-glycosylated site at amino acid 15. Motif-scan results showed that the protein has a casein kinase II phosphorylation site, an N-myristoylation site, and a protein kinase C phosphorylation site.

### Sequence alignment and phylogenetic analysis

**Fig 2:**
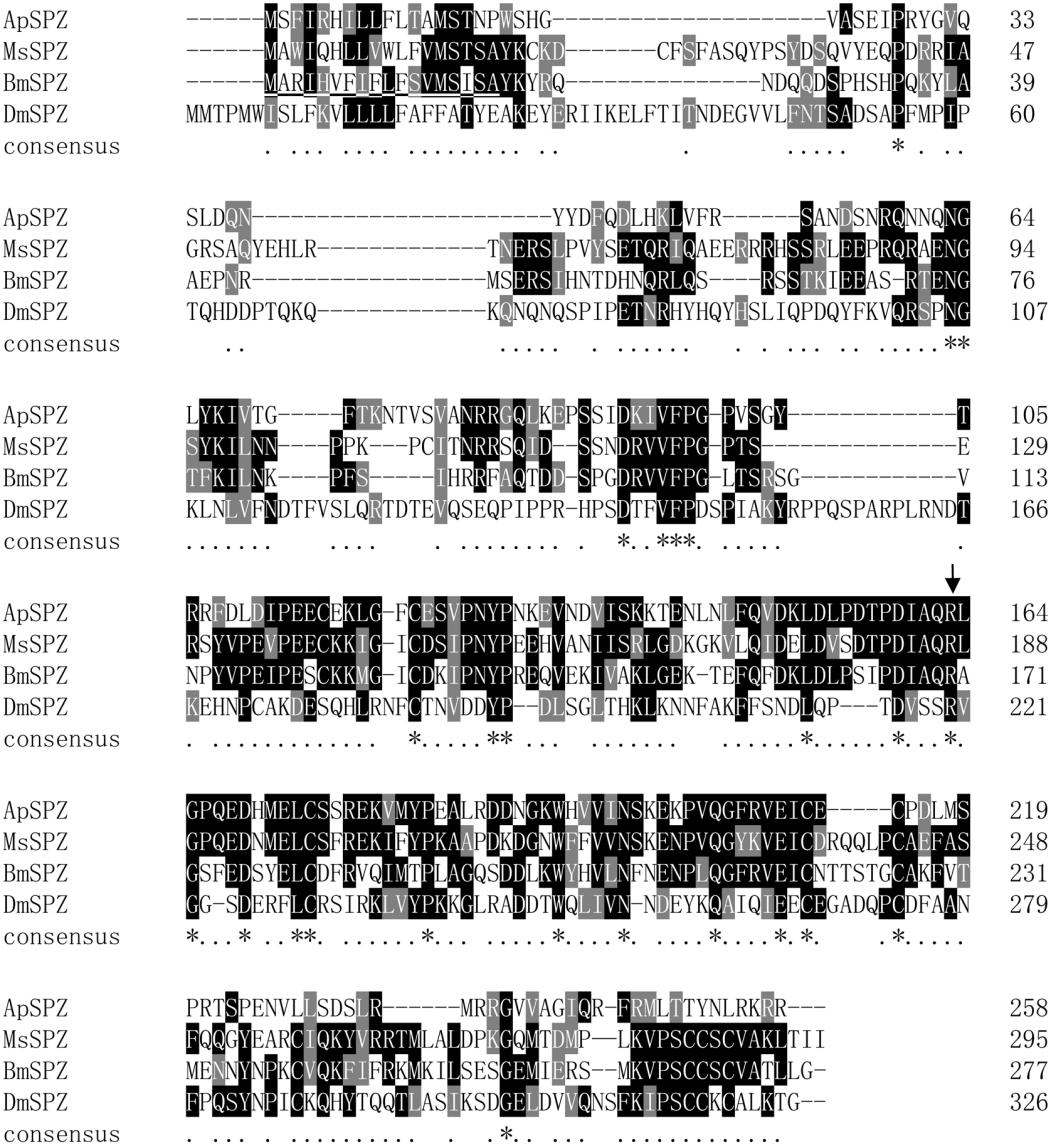
Sequence alignment of spatzles from *A*. *pernyi* (ApSPZ), *M*. *sexta* (MsSPZ), *B*. *mori* and *D*. *melanogaster* (DmSPZ). The amino acid alignment was generated by the Clustal X program. Amino acid residues are shaded as follows based on the conserved percent: 100%, ≥80%, ≥60% and <60% according to the default settings are indicated by white letters on black, white letters on dark grey, white letters on light grey and black letters on white, respectively. The predicted signal peptides of proteins from different organisms are underlined. The P1 residue at the activation cleavage site is completely conserved and is noted with an arrow. The GenBank accession numbers are MsSPZ, ACU68553; BmSPZ, NP_001108066; and DmSPZ, NP_524526.

Homologous alignment of spatzle from *A*. *pernyi* (ApSPZ, KU323402), *Manduca sexta* (MsSPZ, ACU68553), *Drosophila melanogaster* (DmSPZ, NP_524526), and *Bombyx mori* (BmSPZ, NP_001108066) was performed using the Clustal X program (Fig 2). Sequence alignment showed that the amino acid sequence of ApSPZ is most similar to MsSPZ, with 40% identity, and the gene exhibited 33.15% identity with BmSPZ and 13.58% identity with DmSPZ. The putative activation cleavage site of ApSPZ, located after IAQR^163^, is conserved in MsSPZ and BmSPZ. An activating protease could cleave after the conserved residue Arg 163 in ApSPZ, similar to the confirmed cleavages in DmSPZ and MsSPZ [3,35]. The protein structure prediction showed that ApSPZ matched the template structure for 4bv4.1.A (PROTEIN SPAETZLE C-106). Although three Cys residues in the putative carboxyl-terminal active cystine knot domain in ApSPZ are conserved in *M*. *sexta*, *D*. *melanogaster* and *B*. *mori*, four other Cys residues were not found. The results were consistent with *D*. *melanogaster* Spz8.24, indicating that they are not involved in disulfide formation, and ApSPZ might be similar to DmSpz8.24, which is natively unfolded [3,36].

**Fig 3:**
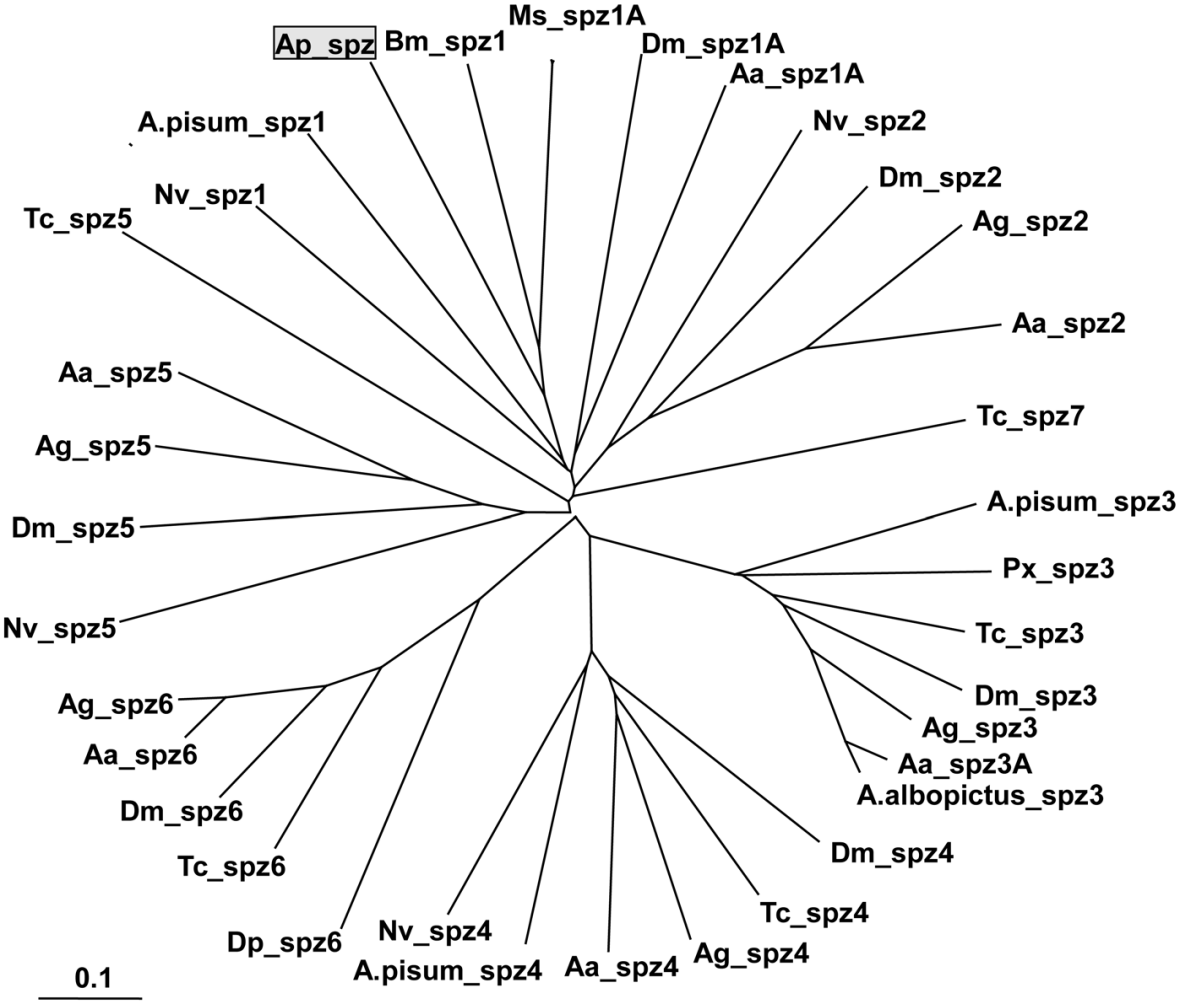
Phylogenetic analysis of the spatzles from *A*. *pernyi* and other insect species. The abbreviations and the GenBank accession numbers of the spatzles are shown in S2 Table.

To assess the phylogenetic relationship between ApSPZ and other insect spatzle proteins, a total of 35 insect spatzle proteins of different spatzle types were collected from the GenBank database (S2 Table). The spatzle amino acid sequences from different insect species were first aligned using Clustal X [33]. The unrooted phylogenetic tree was generated with TreeView Version 1.6.6 [34], and it is shown in Fig 3. ApSPZ was most closely related to BmSPZ1 and MsSPZ1A and was assigned to the SPZ1 group with other insect SPZ1 proteins. Moreover, different types of insect spatzles were assigned to corresponding groups. *Tribolium castaneum* Spatzle5 (TcSPZ5) has a lower degree of sequence conservation with other insect SPZ5 proteins. In conclusion, ApSPZ might be a new member of the spatzle type 1 family within the spatzle superfamily.

### Relative mRNA expression patterns of genes involved in the Toll pathway

**Fig 4:**
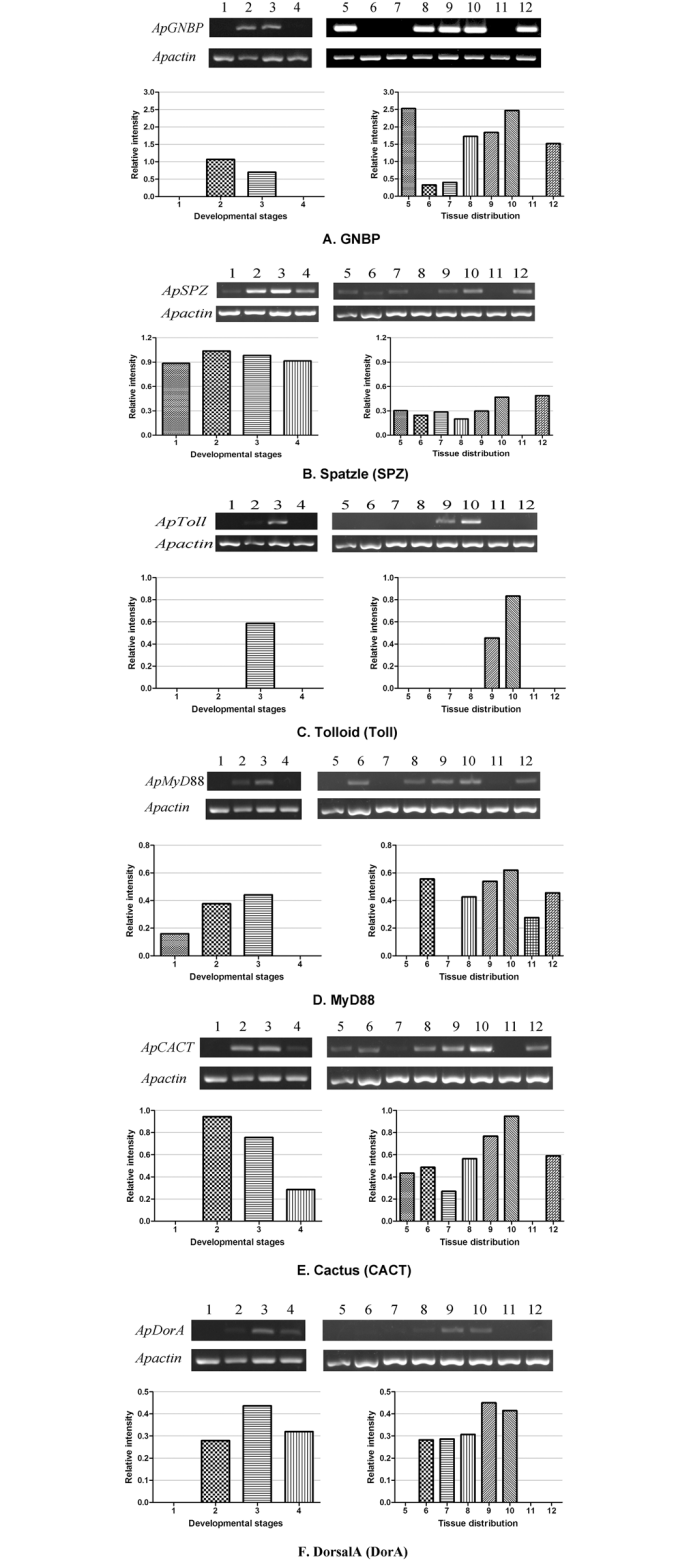
Expression and relative intensity of genes in the Toll pathway during different developmental stages and in different tissues of 5^th^ instar larvae in *A*. *pernyi*. Lane 1, eggs on the 5th day; lane 2, 5th instar larvae; lane 3, pupae; lane 4, moths; lane 5, epidermis; lane 6, silk gland; lane 7, blood; lane 8, gonads; lane 9, Malpighian tubules; lane 10, fat body; lane 11, midgut; lane 12, muscle.

Semi-quantitative RT-PCR was performed to examine the expression of genes involved in the Toll pathway in different developmental stages and various tissues of the *A*. *pernyi* 5th instar larvae (Fig 4). In addition to *A*. *pernyi spatzle*, the genes selected were *A*. *pernyi GNBP* (GenBank accession No. KF725771), *A*. *pernyi MyD88* (GenBank accession No. KF670143), *A*. *pernyi Tolloid* (GenBank accession No. KF670144), *A*. *pernyi cactus* (GenBank accession No. KF670142) and *A*. *pernyi dorsalA* (GenBank accession No. JF488068). To standardize the templates, *Apactin* was used as an internal control [25]. RT-PCR was performed with the specific primer pairs (shown in S1 Table) for the each gene.

As shown in Fig 4A, the *A*. *pernyi GNBP* gene was expressed in larvae and pupae during four developmental stages. *ApGNBP* was expressed in all of the tissues examined except for the midgut, and the highest mRNA levels were found in the epidermis and fat body. GNBP is a pattern recognition protein that enables the host to detect invading bacteria [37]. GNBP and the peptidoglycan recognition protein SA (PGRP-SA) jointly activate the Toll pathway against gram-positive bacterial infections in *Drosophila* [1,38]. *A*. *pernyi spatzle* (*ApSPZ*) was expressed during four developmental stages, including eggs, larvae, pupae and moths, indicating that ApSPZ has an important role throughout the entire life cycle of *A*. *pernyi*. The highest mRNA levels were observed in the larval stage. *ApSPZ* was expressed in all of the tissues examined except the midgut, and the highest mRNA levels were found in the fat body and muscle (Fig 4B). *A*. *pernyi Tolloid* (*ApToll*) mRNA was only expressed in the pupae stage and not in the larvae, which might be due to whole larva sampling. *ApToll* was expressed in Malpighian tubules and the fat body, and the highest mRNA levels were observed in the fat body (Fig 4C). *A*. *pernyi Tolloid* is a Toll family member and could be assigned to the Toll-1 group with *D*. *melanogaster* Tolloid (GenBank accession No. AAF56329). *D*. *melanogaster Tolloid* was detected in blood cells and the fat body, but no transcripts were found in the lymph gland [39]. *A*. *pernyi MyD88* (*ApMyD88*) was expressed in the eggs, larvae and pupae. *ApMyD88* was expressed in all of the tissues examined except for the epidermis and blood, and the highest mRNA levels were found in the fat body (Fig 4D). *A*. *pernyi cactus* (*ApCACT*) was expressed in the larvae, pupae and moths, and *ApCACT* was expressed in all of the tissues examined except for the midgut. Transcript levels were most abundant in the fat body (Fig 4E). The *A*. *pernyi dorsalA* (*ApDorA*) gene was expressed in the larvae, pupae and moths. *ApDorA* was expressed in the silk gland, blood, spermary/ovary, Malpighian tubules and fat body and was not detected in the epidermis, midgut and muscle. Moreover, the highest mRNA levels were found in the Malpighian tubules and the fat body (Fig 4F), which was consistent with a previous study [24].

The genes involved in the Toll pathway were predominantly expressed in immune-responsive fat body tissue, indicating that these genes play a crucial role in *A*. *pernyi* innate immunity. Further studies of these genes are underway to clarify the immune response of *A*. *pernyi* against infection by microorganisms.

### Toll pathway immunity in *A*. *pernyi* against pathogenic microorganisms

To investigate the role of the *A*. *pernyi* Toll signaling pathway in the response to different pathogens, the relative mRNA levels of genes in the Toll pathway were assessed by qRT-PCR after *A*. *pernyi* was challenged by different pathogenic microorganisms.

**Fig 5:**
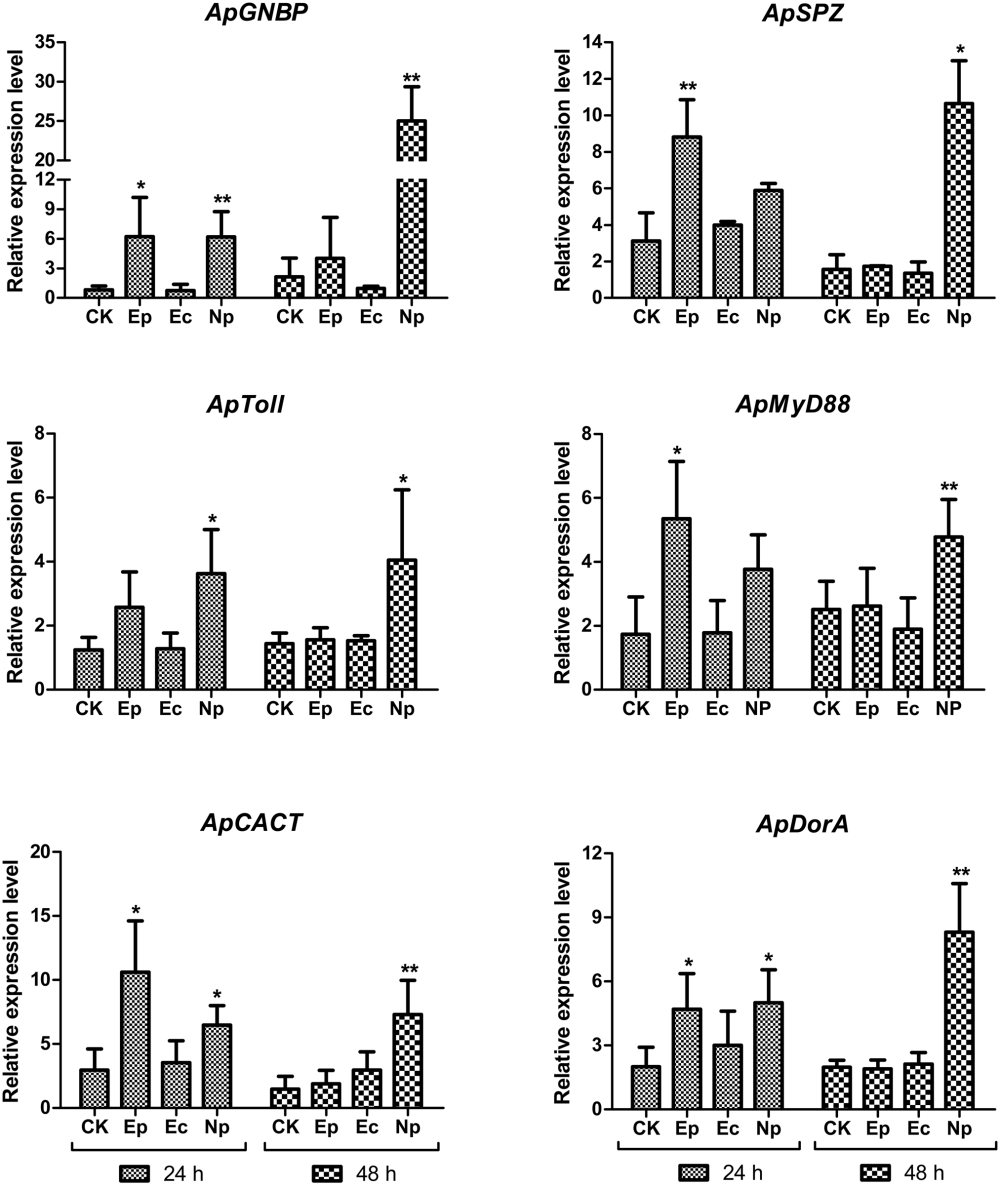
Relative mRNA levels of genes in the Toll pathway were detected by qRT-PCR in *A*. *pernyi* treated with pathogenic microorganisms. The fifth instar larvae were inoculated by oral feeding with *Escherichia coli* (Ec), *Enterococcus pernyi* (Ep), or *Nosema pernyi* (Np) on the first day, and larvae fed sterile water were used as control samples (CK). Fat bodies dissected from each group for RNA extraction 24 h and 48 h after inoculation were used for qRT-PCR testing.

As shown in Fig 5, after infection with different pathogenic microorganisms, significant changes were observed in the transcriptional levels of Toll pathway genes in *A*. *pernyi*.

In this study, the expression levels of *ApGNBP* were significantly elevated in *A*. *pernyi* compared to CK after 24 h of infection with a gram-positive bacterium (Ep) (P<0.05) and fungus (Np) (P<0.01). After infection with Np, *ApGNBP* expression continued to increase, exhibiting a 10-fold change compared to CK at 48 h (P<0.01). However, no significant change was observed in *A*. *pernyi* after infection with *E*. *coli*. The transcriptional levels of *ApSPZ* were obviously increased in *A*. *pernyi* at 24 h (P<0.01) but were similar to CK 48 h (P>0.05) after infection with Ep. The expression levels of *ApSPZ* in *A*. *pernyi* infected with Np were elevated, and there was no significant difference compared to CK at 24 h (P>0.05). However, levels were significantly different compared to CK at 48 h (P<0.05). Meanwhile, no significant differences were observed in *A*. *pernyi* after infection with a gram-negative bacterium (Ec). *ApToll* levels were slightly increased in *A*. *pernyi* at 24 h, but this was not significant (P>0.05), and they were similar to CK 48 h (P>0.05) after infection with Ep. The expression levels of *ApToll* in *A*. *pernyi* infected by Np were increased, and there was a significant difference at 24 h (P<0.05) and 48 h (P<0.05) compared to CK. There was no significant change between *A*. *pernyi* infected with Ec and CK. The levels of *ApMyD88* were significantly increased in *A*. *pernyi* at 24 h (P<0.05) and were similar to CK at 48 h (P>0.05) after infection with Ep. Following infection with Np, *ApMyD88* levels were increased, and they were significantly different at 48 h (P<0.01) compared to CK, although there was no significant change at 24 h (P>0.05). There were no significant differences between *A*. *pernyi* infected with Ec and CK, and the expression level of *ApMyD88* in *A*. *pernyi* infected with Ec was slightly decreased at 48 h. The expression levels of *ApCACT* were significantly elevated in *A*. *pernyi* at 24 h after infection with Ep (P<0.05) and Np (P<0.05), and the effect of Ep on *A*. *pernyi* was greater than that of Np, but no significant difference was observed in *A*. *pernyi* infected with Ec. Forty-eight hours after inoculation, there were significant increases in gene expression only in *A*. *pernyi* infected with Np (P<0.01), whereas other groups showed slightly elevated expression but no significant differences. The expression levels of *ApDorA* were significantly increased in *A*. *pernyi* 24 h after infection with Ep (P<0.05) and Np (P<0.05), and there was a sharp increase 48 h after infection with Np (P<0.01); however, no significant differences were observed in *A*. *pernyi* after infection with Ec.

The expression analysis of Toll pathway genes associated with *A*. *pernyi* orally infected with fungi (Np), gram-positive bacteria (Ep) and gram-negative (Ec) bacteria revealed specific interactions between host immunity and pathogens, indicating that the Toll pathway could be activated by challenge with Np and Ep. However, no significant differences in Toll pathway genes were observed in *A*. *pernyi* after infection with Ec, indicating that the Toll pathway does respond to gram-negative bacteria.

In summary, the Toll signaling pathway in *A*. *pernyi* was activated by fungi and gram-positive bacteria but not by gram-negative bacteria.

## Discussion and Conclusions

The Toll signaling pathway plays a crucial role in the insect innate immune response against gram-positive bacteria, fungi and viruses [1–5]. Although it has been shown that the Toll pathway can be activated to release antimicrobial peptides to defend against microorganism invasion in many insects, there have been no reports investigating the Toll pathway in *A*. *pernyi*. Spatzle is a key protein that only requires an endogenous cytokine ligand for activation and signaling of the Toll pathway. Spatzle is a maternal effect gene that establishes the dorsal-ventral pattern of the *Drosophila* embryo and is required for Toll pathway signaling [40]. Spatzle is also a key signal transducer for immune responses [41]. It has an important function as a ligand for Toll receptors, and spatzle must be cleaved to stimulate Toll-dependent immunity-related gene expression. Moreover, spatzle is processed by trypsin or proteinase to release the cysteine knot domain for interaction with Toll receptors [2,3,42–44]. In this study, cloning, sequencing and expression analysis of the *A*. *pernyi* spatzle (*ApSPZ*) gene were performed. The *ApSPZ* ORF is 777 bp and encodes a 258 amino acids protein. Analysis indicated that ApSPZ is a nuclear and secretory protein. Sequence alignment demonstrated that ApSPZ has a putative activation cleavage site located after IAQR^163^, indicating that an activating protease would cleave after this specific Arg residue, which is similar to DmSPZ [35] and MsSPZ [3]. Only three Cys residues in the putative carboxyl-terminal active cystine knot domain of ApSPZ were conserved compared to spatzles from *M*. *sexta*, *D*. *melanogaster* and *B*. *mori* [3]. The results agree with the structure of *D*. *melanogaster* SPZ8.24, suggesting that they are not involved in disulfide formation. ApSPZ might be similar to DmSpz8.24, which is natively unfolded [36]. Phylogenetic analysis showed that ApSPZ was most closely related to BmSPZ1 and MsSPZ1A, indicating that ApSPZ is a new member of the spatzle type 1 family. Furthermore, the expression patterns of genes involved in the Toll pathway were analyzed, and the results showed that only *ApSPZ* was expressed in all four developmental stages, indicating that the ApSPZ plays an important role throughout the entire life cycle of *A*. *pernyi*. Possibly due to whole larva sampling, all of the genes were detected during the larval stage except for *ApToll*. The tissue distribution indicated that these genes were predominantly expressed in immune-responsive fat body tissue, indicating that these genes play a crucial role in the innate immunity of *A*. *pernyi*. Furthermore, according to the qRT-PCR results, the relative mRNA levels of most genes in the Toll pathway were significantly different from the control 24 h or 48 h after *A*. *pernyi* was challenged by the fungus *N*. *pernyi* and the gram-positive bacterium *E*. *pernyi*. The mRNA levels of these genes were elevated but not significantly different in *A*. *pernyi* after infection with *E*. *coli*, indicating that the Toll pathway does not respond to gram-negative bacteria. These results were consistent with previous studies [45,46].

In conclusion, a novel spatzle belonging to the spatzle type 1 family from *A*. *pernyi* has been identified. *ApSPZ* was expressed during all developmental stages of *A*. *pernyi*. Other genes associated with the Toll pathway were mainly expressed in the fat body, suggesting that the Toll pathway is important in the *A*. *pernyi* innate immune system for defending against pathogenic microorganisms. Moreover, infection of *A*. *pernyi* with the fungus *N*. *pernyi* and the gram-positive bacterium *E*. *pernyi* but not by the gram-negative bacterium *E*. *coli* activates the Toll signaling pathway.

## Supporting Information

S1 Fig Different developmental stages of *A*. *pernyi*. (TIF)

S2 Fig Survival conditions of *A*. *pernyi* larvae. (TIF)

S1 Table Sequences of primers used in this paper. (PDF)

S2 Table Information on spatzles from different insect species used in Fig 3. (PDF)
