# Supplementary figures and images for: The Toll Signaling Pathway in the Chinese Oak Silkworm, Antheraea pernyi: Innate Immune Responses to Different Microorganisms
Source: PLoS One. 2016 Aug 2;11(8):e0160200. doi: 10.1371/journal.pone.0160200 (PMC4970820; doi:10.1371/journal.pone.0160200)

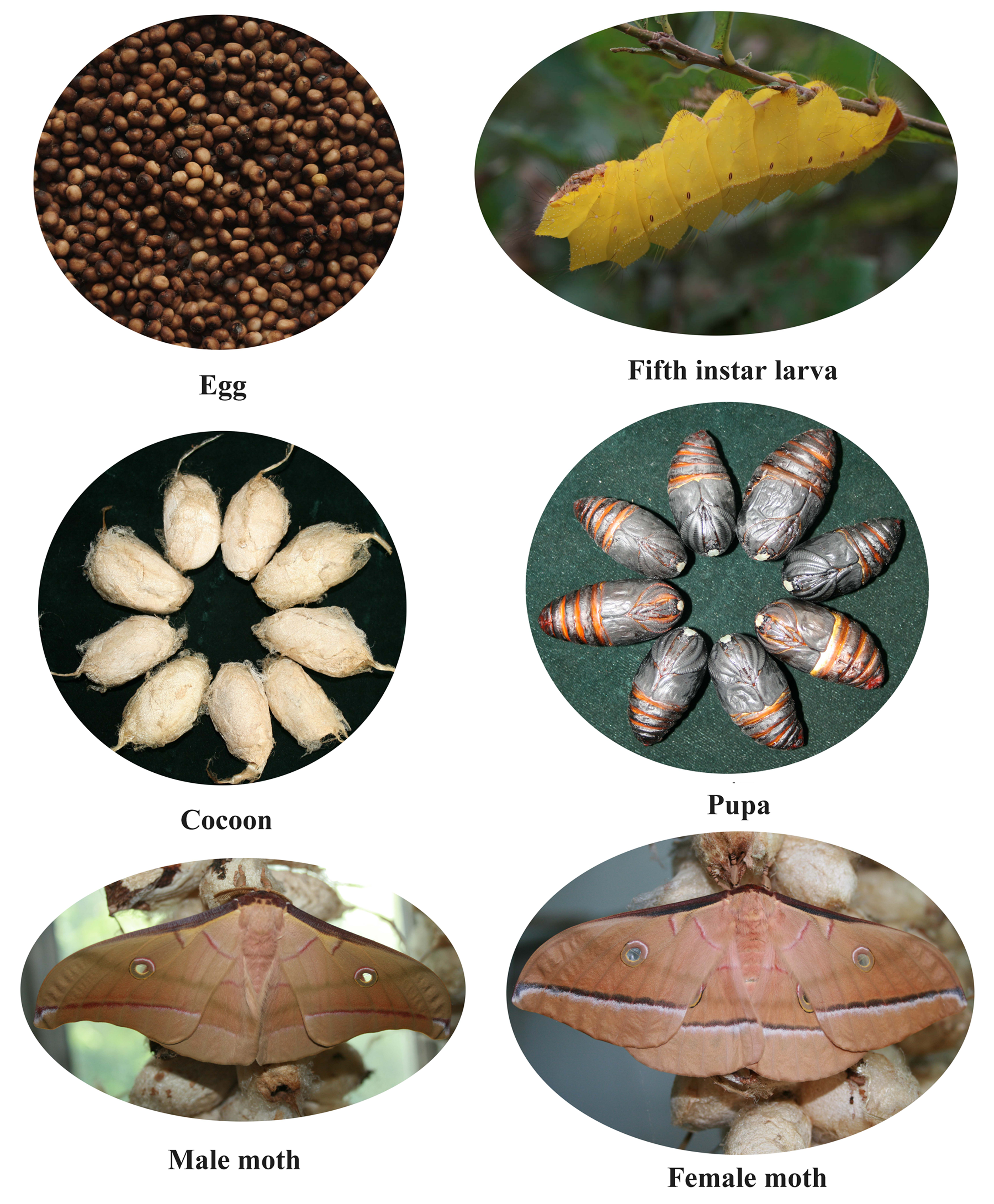

Supplement: S1 Fig — (TIF) [file pone.0160200.s001.tif]

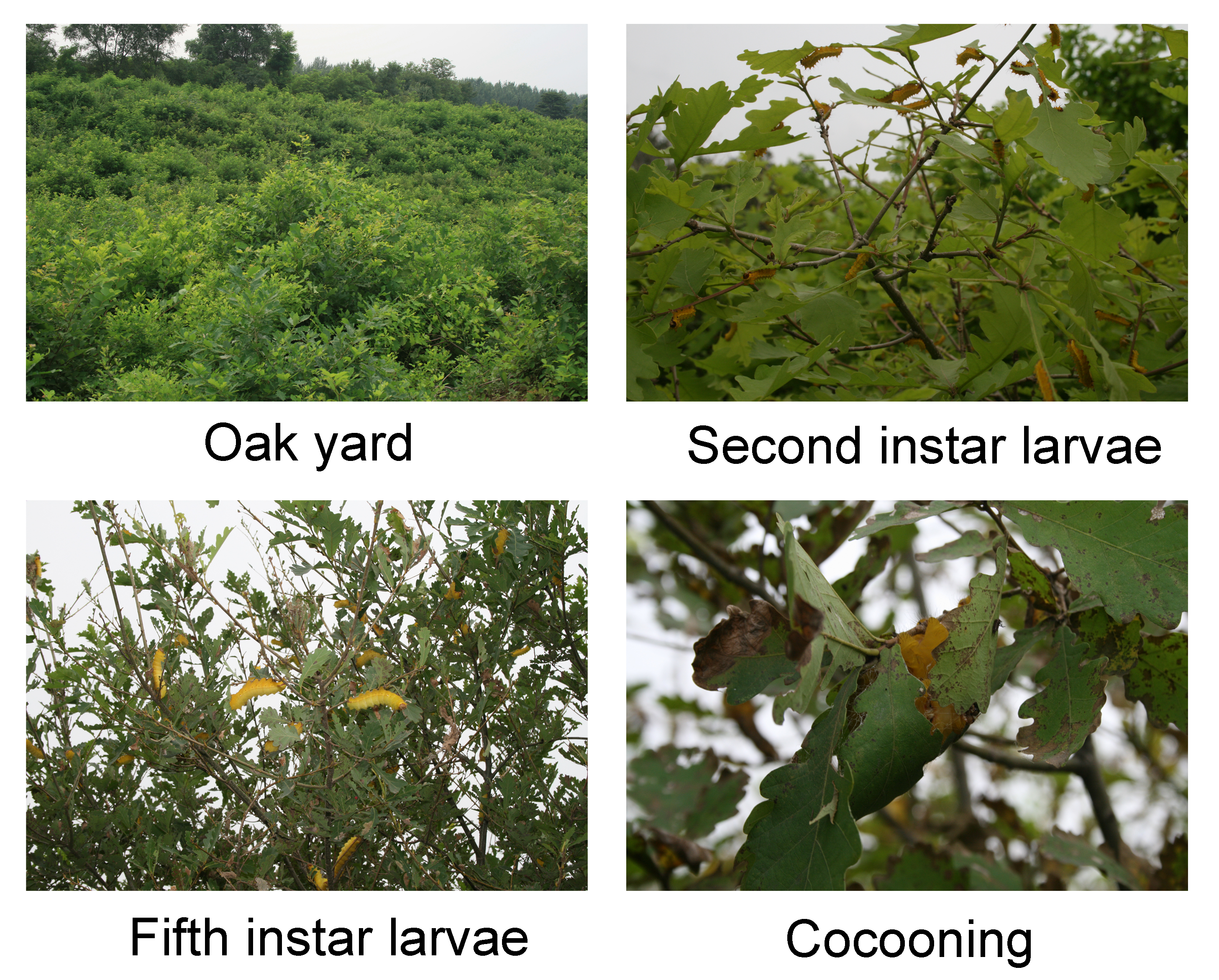

Supplement: S2 Fig — (TIF) [file pone.0160200.s002.tif]
