# Supplementary material for: The Toll Signaling Pathway in the Chinese Oak Silkworm, Antheraea pernyi: Innate Immune Responses to Different Microorganisms
Source: PLoS One. 2016 Aug 2;11(8):e0160200. doi: 10.1371/journal.pone.0160200 (PMC4970820; doi:10.1371/journal.pone.0160200)
Supplement: S1 Table — (PDF) [file pone.0160200.s003.pdf]

Table S1. Sequences of primers used in this paper

Primer type	Primers abbreviations	Sequence (5' to 3')	Gene Name	GenBank Accession No.
RT-PCR	SPZ-F	ATATACACGGCGCTTTGACC	*Antheraea pernyi* Spatzle	KU323402
	SPZ-R	TACATGCCACTTCCCGTTG		
	GNBP-F	AATCCCAACATCGAGCTGAA	*Antheraea pernyi* GNBP	KF725771
	GNBP-R	GTCGCTGGTACGAAACGAAT		
	D88-F	GGCGCAACTAATCTCCAAAC	*Antheraea pernyi* MyD88	KF670143
	D88-R	CAATCTGGGCATAACCCTGT		
	TOLL-F	GATGGATCGGTTCAGAGGAA	*Antheraea pernyi* tolloid	KF670144
	TOLL-R	TTTAGTCCCGCAGAAAGTGC		
	CACT-F	ATTCCGGTTTCCTCTCTGGT	*Antheraea pernyi* cactus	KF670142
	CACT-R	CTGGGCACACCCTGATAAGT		
	DorA-F	GCATCCGAAGTCATCTACGA	*Antheraea pernyi* dorsalA	JF488068
	DorA-R	GTCCGTCAGCCTCTTCAGTT		
qRT-PCR	qSPZ-F	AAATTGGGCTTCTGCGAAT	*Antheraea pernyi* Spatzle	KU323402
	qSPZ-R	TCTGGTGTGTCAGGTAAATCCA		
	qGNBP-F	GGCTCCAGATCGCATAACTC	*Antheraea pernyi* GNBP	KF725771
	qGNBP-R	GAATGAGGCACGTTGCAGTA		
	qD88-F	ACAGGGTTATGCCCAGATTG	*Antheraea pernyi* MyD88	KF670143
	qD88-R	AAGAACTGGCTCTCCGTGAA		
	qTOLL-F	TCTACAAATCGGAGGGATGC	*Antheraea pernyi* tolloid	KF670144
	qTOLL-R	TCTCATCATCGGCCCTTTAC		
	qCACT-F	CCAAAGGCAGTCCGATTTAC	*Antheraea pernyi* cactus	KF670142
	qCACT-R	CTGGGCACACCCTGATAAGT		
	qDorA-F	CCATCGTTGTGTCCTGTGTC	*Antheraea pernyi* dorsalA	JF488068
	qDorA-R	CTCCTCTGTGATCTCGGTACG		
