# Supplementary material for: The Toll Signaling Pathway in the Chinese Oak Silkworm, Antheraea pernyi: Innate Immune Responses to Different Microorganisms
Source: PLoS One. 2016 Aug 2;11(8):e0160200. doi: 10.1371/journal.pone.0160200 (PMC4970820; doi:10.1371/journal.pone.0160200)
Supplement: S2 Table — (PDF) [file pone.0160200.s004.pdf]

Table S2. Information on spatzles from different insect species used in Fig 3

No.	Abbreviations	Species	Spatzle type	GenBank Accession No.
1	Ap_spz	*Antheraea pernyi*	spz	KU323402
2	Aa_spz1	*Aedes aegypti*	spz1	XP_001663627
3	A.pisum_spz1	*Acyrthosiphon pisum*		NP_001153589
4	Ms_spz1A	*Manduca sexta*		ACU68553
5	Dm_spz1A	*Drosophila melanogaster*		NP_524526
6	Bm_spz1	*Bombyx mori*		NP_001108066
7	Nv_spz1	*Nasonia vitripennis*		XP_001606369
8	Aa_spz2	*Aedes aegypti*	spz2	XP_001659184
9	Ag_spz2	*Anopheles gambiae* str. PEST		XP_557166
10	Dm_spz2	*Drosophila melanogaster*		NP_729009
11	Nv_spz2	*Nasonia vitripennis*		XP_001607462
12	Px_spz3	*Papilio xuthus*	spz3	BAM17861
13	A.pisum_spz3	*Acyrthosiphon pisum*		NP_001153591
14	Tc_spz3	*Tribolium castaneum*		NP_001153625
15	Dm_spz3	*Drosophila melanogaster*		NP_609160
16	Ag_spz3	*Anopheles gambiae* str. PEST		XP_317093
17	Aa_spz3A	*Aedes aegypti*		XP_001653322
18	A.albopictus_spz3	*Aedes albopictus*		JAC10774
19	Dm_spz4	*Drosophila melanogaster*	spz4	NP_609504

20	Tc_spz4	*Tribolium castaneum*		EFA09263
21	Ag_spz4	*Anopheles gambiae* str. PEST		XP_317626
22	Aa_spz4	*Aedes aegypti*		ABM68624
23	A.pisum_spz4	*Acyrthosiphon pisum*		NP_001153592
24	Nv_spz4	*Nasonia vitripennis*		XP_001605307
25	Tc_spz5	*Tribolium castaneum*	spz5	EEZ97725
26	Aa_spz5	*Aedes aegypti*		XP_001654338
27	Ag_spz5	*Anopheles gambiae* str. PEST		XP_308593
28	Dm_spz5	*Drosophila melanogaster*		NP_647753
29	Nv_spz5	*Nasonia vitripennis*		XP_001599503
30	Ag_spz6	*Anopheles gambiae* str. PEST	spz6	XP_314007
31	Aa_spz6	*Aedes aegypti*		XP_001655918
32	Dm_spz6	*Drosophila melanogaster*		NP_611961
33	Tc_spz6	*Tribolium castaneum*		NP_001164082
34	Dp_spz6	*Danaus plexippus*		EHJ65295
35	Tc_spz7	*Tribolium castaneum*	spz7	EEZ99267
